# Efficacy and safety of apixaban vs warfarin in patients with atrial fibrillation and prior bioprosthetic valve replacement or valve repair: Insights from the ARISTOTLE trial

**DOI:** 10.1002/clc.23178

**Published:** 2019-04-09

**Authors:** Patricia O. Guimarães, Sean D. Pokorney, Renato D. Lopes, Daniel M. Wojdyla, Bernard J. Gersh, Anna Giczewska, Anthony Carnicelli, Basil S. Lewis, Michael Hanna, Lars Wallentin, Dragos Vinereanu, John H. Alexander, Christopher B. Granger

**Affiliations:** ^1^ Duke Clinical Research Institute Duke University School of Medicine Durham North Carolina; ^2^ Division of Cardiovascular Diseases Mayo Clinic College of Medicine Rochester Minnesota; ^3^ Department of Biomedical Engineering, Faculty of Electronics Telecommunications and Informatics, Gdansk University of Technology Poland; ^4^ Department of Cardiovascular Medicine Lady Davis Carmel Medical Center Haifa Israel; ^5^ Bristol‐Myers Squibb Princeton New Jersey; ^6^ Uppsala Clinical Research Center, Department of Medical Sciences, Cardiology Uppsala University Uppsala Sweden; ^7^ Department of Cardiology University of Medicine and Pharmacy Carol Davila, University and Emergency Hospital Bucharest Romania

**Keywords:** apixaban, atrial fibrillation, bioprosthetic valves, valve repair

## Abstract

**Background:**

The optimal anticoagulation strategy for patients with atrial fibrillation (AF) and bioprosthetic valve (BPV) replacement or native valve repair remains uncertain.

**Hypothesis:**

We evaluated the safety and efficacy of apixaban vs warfarin in patients with AF and a history of BPV replacement or native valve repair.

**Methods:**

Using data from Apixaban for Reduction in Stroke and Other Thromboembolic Events in Atrial Fibrillation (ARISTOTLE) (n = 18 201), a randomized trial comparing apixaban with warfarin in patients with AF, we analyzed the subgroup of patients (n = 251) with prior valve surgery. We contacted sites by telephone to obtain additional data about prior valve surgery. Full data were available for 156 patients. The primary efficacy endpoint was stroke/systemic embolism. The primary safety endpoint was major bleeding. Treatment groups were compared using a Cox regression model.

**Results:**

In ARISTOTLE, 104 (0.6%) patients had a history of BPV replacement (n = 73 [aortic], n = 26 [mitral], n = 5 [mitral and aortic]) and 52 (0.3%) had a history of valve repair (n = 50 [mitral], n = 2 [aortic]). Among patients with BPVs, 55 were randomized to apixaban and 49 to warfarin. Among those with a history of native valve repair, 32 were randomized to apixaban and 20 to warfarin. Overall clinical event rates were low, with no significant differences between apixaban and warfarin for any outcomes.

**Conclusions:**

In patients with AF and a history of BPV replacement or repair, the safety and efficacy of apixaban compared with warfarin was consistent with results from ARISTOTLE. These data suggest that apixaban may be reasonable for patients with BPVs or prior valve repair, though future larger randomized trials are needed.

**ClinicalTrials.gov:**

NCT00412984.

The optimal anticoagulation strategy for patients with atrial fibrillation (AF) and a history of bioprosthetic valve (BPV) replacement or valve repair remains uncertain. Of the major clinical trials of clinically available direct‐acting oral anticoagulants for thromboembolic prevention in AF, only Apixaban for Reduction in Stroke and Other Thromboembolic Events in Atrial Fibrillation (ARISTOTLE) and Effective Anticoagulation with Factor Xa Next Generation in Atrial Fibrillation‐Thrombolysis in Myocardial Infarction 48 (ENGAGE AF‐TIMI 48) included patients with BPVs or valve repair.^1^, ^2^ A pre‐specified subgroup analysis of 191 patients with BPVs from ENGAGE AF‐TIMI 48 revealed similar rates of stroke/systemic embolism and major bleeding among patients treated with edoxaban as compared with warfarin.^3^ In ARISTOTLE, patients with moderate‐to‐severe valvular heart disease (excluding those with moderate‐to‐severe mitral stenosis) or prior valve surgery (native valve repair or BPV replacement) were found to have significantly higher rates of stroke/systemic embolism and mortality than patients without known valvular heart disease, as well as numerically (although not statistically significant) higher rates of bleeding.^5^ Despite this higher risk, there was no evidence of effect modification for the benefits of apixaban over warfarin with regard to stroke/systemic embolism, major bleeding, and all‐cause mortality.^4^, ^5^ However, subgroup analyses including patients with prior valve repair or replacement were not performed. We aimed to evaluate the safety and efficacy of apixaban vs warfarin in the subgroup of patients from ARISTOTLE with a history of BPV replacement or native valve repair.

## 1 METHODS

ARISTOTLE (NCT00412984) included patients with AF or atrial flutter and ≥1 risk factors for stroke: age ≥75 years, previous stroke/transient ischemic attack, symptomatic heart failure, diabetes, or hypertension.^4^ Patients were randomized to receive apixaban 5 mg twice daily or dose‐adjusted warfarin, with a target international normalized ratio of 2.0 to 3.0. A reduced dose of apixaban (2.5 mg twice daily) was given to patients who met ≥2 of the following criteria: age ≥ 80 years, body weight ≤60 kg, serum creatinine ≥1.5 mg/dL. Patients with a history of BPV replacement or native valve repair (valvuloplasty, annuloplasty, commissurotomy) were eligible for inclusion. Those with moderate or severe mitral stenosis or mechanical heart valves were excluded. All patients provided written informed consent, and approval was received from appropriate ethics committees at participating sites.

The initial ARISTOTLE case report form collected binary data indicating whether patients had a history of valve surgery and which valve was involved. For our study, ARISTOTLE sites were contacted by telephone to obtain additional data including date, type, and anatomic location of prior valve surgery. In patients with prior aortic valve surgery for whom confirmatory data were unable to be obtained from a study site, the surgery was assumed to be BPV replacement (n = 57) because of the low prevalence of aortic valve repair in clinical practice. Patients without study site confirmation of non‐aortic valve surgery (n = 90), with mechanical replacement (n = 4), or right‐sided valve repair only (n = 1) were excluded.

Efficacy outcomes included stroke or systemic embolism, all‐cause stroke, ischemic stroke, myocardial infarction, all‐cause death, and cardiovascular death. Safety outcomes included major bleeding, major, or clinically relevant non‐major bleeding, intracranial hemorrhage, gastrointestinal bleeding, and any bleeding. Study outcomes were adjudicated by an independent committee blinded to study drug assignment. Baseline characteristics of patients with BPVs or native valve repair treated with apixaban or warfarin were compared using Wilcoxon tests for continuous variables and *χ* ^2^ tests for categorical variables. Clinical outcomes of randomized treatment groups were compared using a Cox regression model. The median (25th, 75th) durations of follow‐up for patients with BPVs and a history of native valve repair were 1.6 (1.3, 2.2) and 1.8 (1.4, 2.3) years.

## 2 RESULTS

Of 18 201 patients enrolled in ARISTOTLE, 251 patients had a history of valve surgery. A total of 95 patients were excluded because lack of study site confirmation of non‐aortic valve surgery (n = 90), presence of mechanical valve (n = 4), or right‐sided valve repair only (n = 1). Confirmatory data were unable to be obtained from a study site in 57 patients, thus, valve surgery was assumed to be BPV replacement as described above.

Of the remaining 156 patients, 104 (0.6%) had a history of bioprosthetic replacement (n = 73 [aortic], n = 26 [mitral], n = 5 [mitral and aortic]), and 52 (0.3%) had a history of native valve repair (n = 50 [mitral], n = 2 [aortic]).

Among patients with BPVs or native valve repair, 87 were randomized to apixaban and 69 to warfarin. Baseline characteristics of ARISTOTLE patients with BPVs or native valve repair stratified by randomized treatment are presented in Table 1. Median age was 74 years and 39.1% were female. No significant differences were observed between groups for any of the baseline characteristics analyzed. More patients in the apixaban group had prior stroke, transient ischemic attack, or systemic embolism than those in the warfarin group (27.6% vs 17.4%), but that difference was not significant. Overall clinical event rates were low (Table 2), with no significant differences between apixaban and warfarin for any outcomes.

**Table 1:** Baseline characteristics among patients with bioprosthetic valves or history of valve repair treated with apixaban or warfarin

Characteristic	Apixaban(N = 87)	Warfarin(N = 69)	*P*‐value
Age, median (25th, 75th), years	72 (63, 79)	74 (65, 78)	0.5088
Female sex, no. (%)	34 (39.1%)	27 (39.1%)	0.9949
BMI, median (25th, 75th), kg/m^2^	26 (24, 31)	27 (24, 32)	0.3879
Prior stroke, TIA, or SE, no. (%)	24 (27.6%)	12 (17.4%)	0.1333
LVEF, no. (%)			0.7243
Normal	50 (58.8%)	41 (62.1%)	
Mild dysfunction	18 (21.2%)	12 (18.2%)	
Moderate dysfunction	10 (11.8%)	10 (15.2%)	
Severe dysfunction	7 (8.2%)	3 (4.5%)	
Diabetes, no. (%)	19 (21.8%)	17 (24.6%)	0.6803
Hypertension, no. (%)	68 (78.2%)	64 (92.8%)	0.0121
Coronary artery disease, no. (%)	36 (41.4%)	32 (46.4%)	0.5319
Prior MI, no. (%)	16 (18.4%)	10 (14.5%)	0.5164
Heart failure, no. (%)	30 (34.5%)	24 (34.8%)	0.9688
Prior bleeding, no. (%)	25 (28.7%)	19 (27.5%)	0.8687
History of falls, no. (%)	4 (4.9%)	3 (4.9%)	1.0000
Type of AF, no. (%)			0.9123
Paroxysmal	17 (19.5%)	13 (18.8%)	
Non‐paroxysmal	70 (80.5%)	56 (81.2%)	
HAS‐BLED score, no. (%)			0.8891
0–1	24 (27.6%)	18 (26.1%)	
2	32 (36.8%)	28 (40.6%)	
≥3	31 (35.6%)	23 (33.3%)	
CHADS_2_ score, no. (%)			0.3008
≤1	31 (35.6%)	18 (26.1%)	
2	26 (29.9%)	28 (40.6%)	
≥3	30 (34.5%)	23 (33.3%)	
Chronic renal disease, no. (%)	3 (3.5%)	5 (7.2%)	0.4681
eGFR, median (25th, 75th)	65 (55, 86)	68 (47, 83)	0.6376
NT‐proBNP, median (25th, 75th), ng/L	707 (448, 1159)	826 (404, 1177)	0.8198
Concomitant medication, no. (%)			
Aspirin	24 (27.6%)	25 (36.2%)	0.2479
Clopidogrel	2 (2.3%)	2 (2.9%)	1.0000
Digoxin	29 (34.5%)	23 (34.3%)	0.9800
ACE inhibitor or ARB	68 (78.2%)	53 (76.8%)	0.8410

Abbreviations: ACE, angiotensin converting enzyme; AF, atrial fibrillation; ARB, angiotensin receptor blocker; BMI, body mass index; eGFR, estimated glomerular filtration rate; LVEF, left ventricular ejection fraction; MI, myocardial infarction; NT‐proBNP, N‐terminal pro‐brain natriuretic peptide; SE, systemic embolism; TIA, transient ischemic attack.

**Table 2:** Clinical events rates among patients with bioprosthetic valves or history of valve repair treated with apixaban or warfarin

Event	Apixaban(N = 87)	Warfarin(N = 69)	HR (95% CI)	*P*‐value
	Rate (n)	Rate (n)		
Stroke or SE	2.77 (4)	1.64 (2)	1.714 (0.313‐9.372)	0.53
All‐cause stroke	2.77 (4)	1.64 (2)	1.714 (0.313–9.372)	0.53
Ischemic or unspecified stroke	2.77 (4)	0.82 (1)	3.286 (0.367‐29.400)	0.29
MI	0.68 (1)	0.81 (1)	0.825 (0.052‐13.194)	0.89
All‐cause death	4.61 (7)	4.79 (6)	1.017 (0.341‐3.037)	0.98
Cardiovascular death	1.32 (2)	1.60 (2)	0.872 (0.123‐6.201)	0.89
Major bleeding	5.87 (7)	6.44 (7)	0.882 (0.309‐2.519)	0.82
Major or CRNM bleeding	7.68 (9)	9.50 (10)	0.781 (0.317‐1.925)	0.59
Intracranial bleeding	0.80 (1)	1.82 (2)	0.467 (0.042‐5.187)	0.54
Gastrointestinal bleeding	2.36 (3)	1.83 (2)	1.244 (0.208‐7.448)	0.81
Any bleeding	32.79 (30)	36.62 (28)	0.866 (0.517‐1.451)	0.59
Stroke or SE/major bleeding	8.18 (11)	6.95 (8)	1.150 (0.462‐2.860)	0.76
Stroke or SE/major bleeding/all‐cause death	11.90 (16)	11.29 (13)	1.051 (0.505‐2.186)	0.90

Abbreviations: CI, confidence interval; CRNM, clinically relevant non‐major; HR, hazard ratio; MI, myocardial infarction; SE, systemic embolism.

## 3 DISCUSSION

Our results in patients with BPVs or history of valve repair are consistent with results from the main ARISTOTLE trial, which showed that apixaban is safe and effective for patients with AF. Despite the limitations of our analysis, which include a small sample size and a low number of events, these are the only data available for apixaban vs warfarin for patients with prior valve surgery. These data, along with similar data from ENGAGE AF‐TIMI 48 showing comparable safety and efficacy of edoxaban compared with warfarin, suggest that non‐vitamin K antagonist oral anticoagulants may be reasonable for the prevention of thromboembolism in patients with AF and prior valve surgery. Larger randomized controlled trials are needed to definitively assess the safety and efficacy of non‐vitamin K antagonist oral anticoagulants in this high‐risk population.

## CONFLICT OF INTEREST

Guimarães, Wojdyla, Giczewska, Carnicelli: None. Pokorney: Research grants: Food and Drug Administration, Bristol‐Myers Squibb, Pfizer, Janssen, Gilead, Boston Scientific; Consultant/advisory board: Boston Scientific and Medtronic. Lopes: Research grants: Bristol‐Myers Squibb, GlaxoSmithKline, Medtronic, Pfizer; Consultant: Bayer, Boehringer Ingelheim, Bristol‐Myers Squibb, Daiichi Sankyo, GlaxoSmithKline, Medtronic, Merck, Pfizer, Portola. Gersh: Consultant: Xenon Pharmaceuticals; Data safety monitoring board: Armetheon, Baxter, CardioVascular Research Foundation, Janssen, Medtronic, Mount Sinai St. Luke's, Teva Pharmaceuticals, Thrombosis Research Institute; Other: Boston Scientific, Cipla Limited, Janssen, St. Jude Medical. Lewis: Institutional research grants: Bristol‐Myers Squibb, Pfizer, Bayer Healthcare; Consultant: Bristol‐Myers Squibb, Pfizer.

Hanna: Employee of Bristol‐Myers Squibb at the time of study conduct. Wallentin: Research grants: AstraZeneca, Boehringer Ingelheim, Bristol‐Myers Squibb/Pfizer, GlaxoSmithKline, Merck/Schering‐Plow, Roche Diagnostics; Consultant: Abbott, AstraZeneca, Boehringer Ingelheim, Bristol‐Myers Squibb/Pfizer, GlaxoSmithKline. Vinereanu: Research grants: Bristol‐Myers Squibb/Pfizer, Boehringer Ingelheim, Janssen/Bayer, Daiichi Sankyo, Novartis, Servier; Consultant: Bristol‐Myers Squibb/Pfizer, Boehringer Ingelheim, Janssen/Bayer, Novartis, and Servier. Alexander: Research grants: AstraZeneca, Bristol‐Myers Squibb, Boehringer Ingelheim, CryoLife, CSL Behring, US Food and Drug Administration, National Institutes of Health, Sanofi, Tenax Therapeutics; Consultant: Cempra, CryoLife, CSL Behring, Merck, Novo Nordisk, Pfizer, Portola, VA Cooperative Studies, VasoPrep Surgical, Zafgen. Granger: Research grants: Armetheon, AstraZeneca, Bayer, Boehringer Ingelheim, Bristol‐Myers Squibb, Daiichi Sankyo, GlaxoSmithKline, US Food and Drug Administration, Janssen, Medtronic Foundation, Novartis, Pfizer; Consultant: Abbvie, Armetheon, AstraZeneca, Bayer, Boehringer Ingelheim, Boston Scientific, Bristol‐Myers Squibb, Daiichi Sankyo, Gilead Sciences, Janssen, Medscape, Medtronic, Merck, National Institutes of Health, Novartis, Pfizer, Sirtex, Verseon.
